# Sex chromosomal dimorphisms narrated by X-chromosome translocation in a spiny frog (*Quasipaa boulengeri*)

**DOI:** 10.1186/s12983-018-0291-8

**Published:** 2018-11-27

**Authors:** Xiuyun Yuan, Yun Xia, Xiaomao Zeng

**Affiliations:** 1 Chengdu Institute of Biology, Chinese Academy of Sciences, No. 9 Section 4, Renmin Nan Road, Chengdu, 610041 China; 2 University of Chinese Academy of Sciences, Beijing, 100049 China

**Keywords:** Sex-linked locus, Sex chromosomal dimorphisms, Whole-chromosome painting, X-chromosome translocation

## Abstract

**Background:**

In the general model of sex chromosome evolution for diploid dioecious organisms, the Y (or W) chromosome is derived, while the homogametic sex presumably represents the ancestral condition. However, in the frog species *Quasipaa boulengeri*, heteromorphisms caused by a translocation between chromosomes 1 and 6 are not related to sex, because the same heteromorphic chromosomes are found both in males and females at the cytological level. To confirm whether those heteromorphisms are unrelated to sex, a sex-linked locus was mapped at the chromosomal level and sequenced to identify any haplotype difference between sexes.

**Results:**

Chromosome 1 was assigned to the sex chromosome pair by mapping the sex-linked locus. X-chromosome translocation was demonstrated and confirmed by the karyotypes of the progeny. Translocation heteromorphisms were involved in normal and translocated X chromosomes in the rearranged populations. Based on phylogenetic inference using both male and female sex-linked haplotypes, recombination was suppressed not only between the Y and normal X chromosomes, respectively the Y and translocated X chromosomes, but also between the normal and translocated X chromosomes. Both males and females shared not only the same translocation heteromorphisms but also the X chromosomal dimorphisms in this frog*.*

**Conclusions:**

The reverse of the typical situation, in which the X is derived and the Y has remained unchanged, is known to be very rare. In the present study, X-chromosome translocation has been known to cause sex chromosomal dimorphisms. The X chromosome has gone processes of genetic differentiation and/or structural changes by chance, which may facilitate sex chromosome differentiation. These sex chromosomal dimorphisms presenting in both sexes may represent the early stages of sex chromosome differentiation and aid in understanding sex chromosome evolution.

**Electronic supplementary material:**

The online version of this article (10.1186/s12983-018-0291-8) contains supplementary material, which is available to authorized users.

## Background

The heterogametic Y or W is usually interpreted as derived, while the homogametic sex represents the ancestral condition [1–3]. The differentiation of heteromorphic sex chromosomes is involved in sexually antagonistic mutations, recombination suppression, chromosome rearrangements, accumulation of repetitive DNA and loss-of-function mutations [4]. The initial step is the acquisition of the sex-determining gene/locus when a pair of homologous autosomes becomes proto-sex chromosomes. In the second step, mutations advantageous for the heterogametic sex but deleterious for the homogametic sex accumulate and promote recombination suppression on the proto-sex chromosomes [4, 5]. A lack of recombination results in the accumulation of loss-of-function mutations and repetitive DNA sequences on the Y or W chromosome [6]. Mechanisms involving chromosome rearrangements or heterochromatization may promote sex chromosome differentiation [7, 8]. The evolutionary outcome of this process is the heteromorphism of sex chromosomes, with the Y or W chromosome differentiated by morphological variations, while the X or Z chromosome has remained unchanged [5, 9]. Heteromorphic Y or W chromosomes exist across the vertebrates; this conservation is common in mammals and birds but has been documented in a limited number of cases in amphibians, fishes, and non-avian reptiles [10–13].

Chromosomal heteromorphy is usually related to sex, as is the case in heteromorphic sex chromosome systems. In the spiny frog species *Quasipaa boulengeri*, however, heteromorphisms have been found in autosomal but not sex-related [14]. In this frog, variant heteromorphisms resulted from a reciprocal translocation between chromosomes 1 and 6, yielding two pairs of heteromorphic chromosomes. At least five different karyomorphs (types I-V) have been observed in natural populations. Unlike the normal karyotype (type I), consisting of two pairs of homomorphic metacentric chromosomes 1 and 6 (MM/mm), all the rearranged karyotypes (types II-V) presented heteromorphic chromosomes. Karyomorph type II (MM/mSt) has a heteromorphism on chromosome 6; in both types III (MT/mm) and V (MT/StSt), it lies on chromosome 1, while the type IV karyomorph was designated as the translocation heterozygote and had two pairs of heteromorphic chromosomes, 1 and 6 (MT/mSt). Notably, these visible heteromorphisms at the cytological level were not directly related to sex, as the same heteromorphic chromosomes were found in both males and females [14].

Recently, a sex-specific microsatellite marker, B08, was identified and amplified in a large number of individuals, providing the evidence for a XX/XY sex system in the frog *Q. boulengeri* [15]. This finding provides a chance to confirm whether the observed heteromorphisms are unrelated to sex. In the present study, after attempting to map the sex-linked marker B08 at the chromosomal level, we found it located on chromosome 1. Therefore, chromosome 1 should be the sex chromosome pair. However, these chromosomal heteromorphisms were seemingly unrelated to sex at the cytological level. One possible reason was that reciprocal translocation had occurred on one of the X chromosomes in females; hence, two kinds of X chromosomes should be present in the rearranged populations: normal and translocated X chromosomes. Through phylogenetic analysis, we confirmed the one Y and two X haplotype groups existed and that recombination was suppressed not only between the Y and normal X chromosomes but also the Y and translocated X chromosomes. Both males and females shared not only the same translocation heteromorphisms but also the same X chromosomal dimorphisms in this frog*.*

## Methods

### Animal sampling

In total, 74 *Q. boulengeri* adults were sampled from 13 populations along the western Sichuan basin of southern China, including 24 individuals (12 ♀ and 12 ♂) with normal karyotype and 50 individuals (33 ♀ and 17 ♂) with rearranged karyotypes (Table 1). Mitotic metaphases were prepared by the bone marrow technique described by Schmid et al. [16]. The karyotype criteria were based on the cytological studies of Qing et al. [14]. All frogs were captured during the breeding season so that males could be identified by their secondary sexual characteristics (spiny belly). Additionally, males and females were distinguished by gonad dissection. The liver tissue was collected and stored in 95% ethanol at − 20 °C until DNA extraction. Genomic DNA was extracted from the liver tissue by a salting-out method [17]. Tissue samples of ~ 2 mm^2^ were incubated overnight at 55 °C in 600 μL lysis buffer (10 mM Tris/HCl; 100 mM NaCl; 100 mM EDTA; 1% SDS; 0.5 mg/mL proteinase K). Following digestion and centrifugation, 180 μL of 5 M NaCl was added to the supernatant. Samples were chilled at − 20 °C for 10 min before centrifugation. The supernatant was transferred to a new tube and DNA was precipitated with isopropyl alcohol. After washing with ethanol, DNA pellets were dissolved in 100 μL ddH_2_O. All animal work in the present study was conducted according to the relevant national and international guidelines. All animal care and experimental procedures were approved by the Chengdu Institute of Biology Animal Care and Use Committee [CIB2015003]. All voucher specimens were deposited in the Herpetological Museum of Chengdu Institute of Biology, Chinese Academy of Sciences.

**Table 1:** Sample sizes of 13 populations of *Quasipaa boulengeri* in the western Sichuan basin

Population	Localities	Longitude	Latitude	Total	NF	NM	RF	RM
1-DYYEC	Yan’e village, Dayi, Sichuan, China	103.4493	30.7080	22	5	5	7	5
2-QLTTS	Mt. Tiantai, Sichuan, China	103.1128	30.2775	18	5	5	7	1
3-PZLMS	Longmenshan town, Pengzhou, Sichuan, China	103.8073	31.2438	2	–	–	2	0
4-DJYHK	Hongkouxiang, Dujiangyan, Sichuan, China	103.6520	31.1204	3	–	–	2	1
5-PZCF	Cifeng town, Pengzhou, Sichuan, China	103.8001	31.1056	4	–	–	2	2
6-WCYX	Yingxiu town, Wenchuan, Sichuan, China	103.3969	31.0236	4	–	–	2	2
7-WCXK	Xuankou town, Wenchuan, Sichuan, China	103.4589	30.9682	3	–	–	2	1
8-QCS	Mt. Qingcheng, Sichuan, China	103.3929	30.9260	3	–	–	2	1
9-DYHP	Hepingxiang, Dayi, Sichuan, China	103.4284	30.7195	4	–	–	2	2
10-DYWS	Wushanxiang, Dayi, Sichuan, China	103.4029	30.6603	3	–	–	3	0
11-DYGTS	Gaotang Temple, Dayi, Sichuan, China	103.4658	30.5847	4	–	–	2	2
12-QLDZ	Daozuoxiang, Qionglai, Sichuan, China	103.2558	30.3167	3	2	1	–	–
13-EMS	Mt. Emei, Sichuan, China	103.2883	29.5837	1	–	1	–	–
Total				74	12	12	33	17

*NF* Number of females with normal karyotype, *NM* Number of males with normal karyotype, *RF* Number of females with rearranged karyotypes, *RM* Number of males with rearranged karyotypes

### Mapping the sex-linked marker at the chromosomal level

The sex-linked locus B08 was mapped at the chromosomal level using the same protocol as described by Yuan et al. [18] with slight modification. Briefly, we collected ten copies of chromosome 1 from metaphase spreads using mechanical microdissection, and we amplified chromosomal DNA sequences with the Discover-sc Single Cell Kit V2 (Vazyme Co., China). Using the isolated DNA as templates, the sex-linked marker was amplified through PCR using the primers B08-F7 (5′- GCACGTACATTCATTCTGTG-3′) and B08-R (5’-TACATACAAGTAGGGGGCTC-3′) from Yuan et al. [15]. The DNA of all chromosomes except 1 were used as negative controls. To ensure identical product size, positive controls were set using genomic DNA. If the marker could be amplified successfully, it was considered to be localized to this chromosome.

### Chromosome probe construction and whole-chromosome painting

To preserve the accuracy of the microdissected chromosomes and single-cell whole-genome amplification technique, whole-chromosome painting for the normal karyotype (type I) was performed as described below. In addition, translocation heterozygotes (type IV) were also used for whole-chromosome painting to investigate the origin of reciprocal translocation. The painting probes of chromosome 1 were labelled with Dig-11-dUTP (Roche Diagnostics, Germany) by a second round of single-chromosome whole-genome amplification (WGA3 kit, Sigma-Aldrich, USA). Painting FISH was accomplished following the protocol of Yang et al. [19] with minor modifications. The chromosome slides were hardened at 55 °C for 2.5 h, treated with 0.01% pepsin in 0.01 N hydrochloric acid at 37 °C for 20 min, denatured in 75% formamide in 2 × SSC at 80 °C for 3 min, and dehydrated in a 70, 90, and 100% ethanol series for 10 min at − 20 °C, followed by air-drying. For blocking highly repetitive DNA, C_0_t-1 DNA of *Q. boulengeri* was made according to the procedure described by Zwick et al. [20]. The hybridization solution contained ~ 200 ng probe DNA, ~ 1500 ng C_0_t-1 DNA, 10% dextran sulfate and 50% formamide in 2 × SSC. The mixture was denatured at 100 °C for 10 min and pre-annealed at 37 °C for 80 min, then added to the abovementioned treated slides and covered with a coverslip and parafilm. After hybridizing overnight in a wet chamber at 37 °C, the probes were detected with anti-digoxigenin-fluorescein Fab fragments (Roche, Germany). The chromosomes were counterstained with 4′, 6-diamidino-2-phenylindole (DAPI, Vector Laboratories, USA). Visualization of probe signals was performed under a digital camera (Leica DFC490) attached to a Leica DM2500 fluorescent photomicroscope. Images were processed using the Adobe Photoshop CS6 program.

### PCR amplification, cloning, sequencing and data analyses

A ~ 400 bp fragment of the B08 locus was amplified using the primers B08-F7 and B08-R as described above. Each of the 50 μL PCR reactions contained 25 μL of 2 × EasyTaq SuperMix (TransGen Biotech, Beijing, China), 0.4 μM of each primer, and 1–2 μL of genomic DNA. The PCR protocol involved an initial denaturation at 94 °C for 180 s, followed by 30 cycles of 94 °C for 30 s, 60 °C for 30 s, and 72 °C for 45 s and a final extension at 72 °C for 8 min. Negative controls were run for all amplifications. Amplified products were visualized on 2% agarose gel stained by ethidium bromide and purified using the TIANgel Midi Purification Kit (TianGen Biotech, Beijing, China). Purified products were cloned using *Escherichia coli* DH5α (TransGen Biotech, Beijing, China) competent cells, using the pMD19-T vector (Takara, Japan) according to the manufacturer’s instructions. Positive clones were sequenced by Sangon Biotech Ltd. Co. (Shanghai, China).

Nucleic acid sequences were checked manually using BioEdit version 7.0.9 [21] and aligned with MEGA 7.0 [22]. These sequences were deposited in GenBank under the accession numbers MH607477–MH607598 (Additional file 1: Table S1). To confirm accurate amplification and observed length differences, we compared the sequence data with results from microsatellite genotyping. A neighbour-joining (NJ) tree was constructed using these sequences in MEGA 7.0.

## Results

### Sex chromosomes

**Fig. 1:**
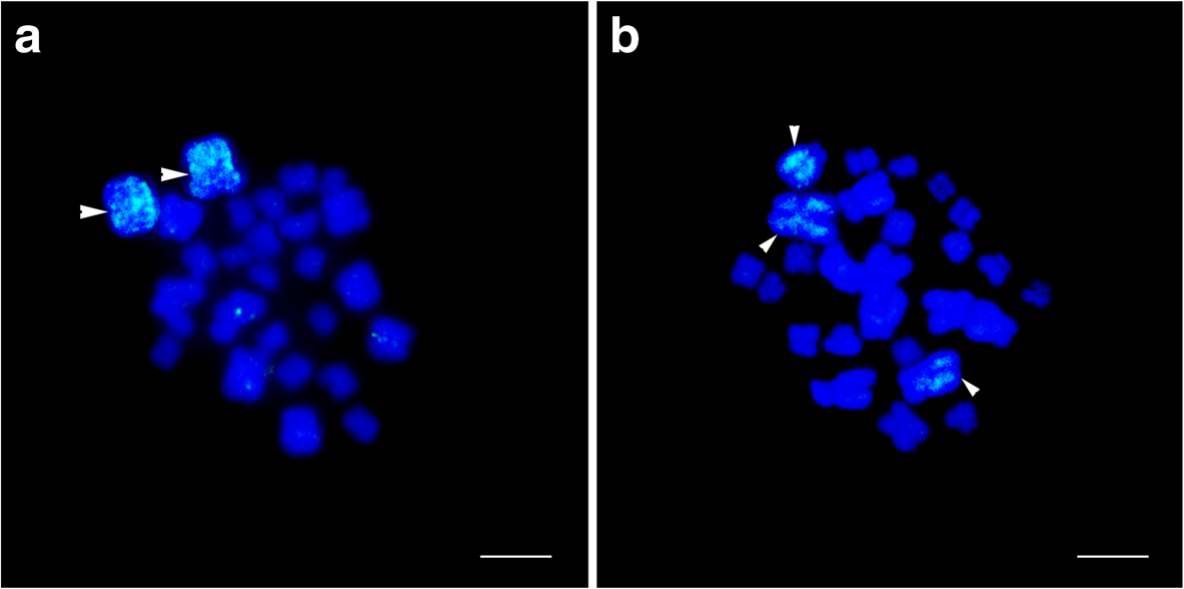
Chromosome painting of normal karyotype (type I) and translocation heterozygotes (type IV) in *Quasipaa boulengeri*. Scale bar = 5 μm. **a** type I, fluorescent signals were detected only in the largest pair of homomorphic chromosome 1 (white arrows). **b** type IV, fluorescent signals were observed in the largest metacentric chromosome 1, the telocentric chromosome including part of chromosome 1, and a large sub-telocentric chromosome 6 (white arrows)

After we repeated the experiment three times for different chromosome 1 sets, the sex-linked locus B08 was unambiguously assigned to chromosome 1. The size of the PCR products was consistent with the positive control groups in which genomic DNA acted as the DNA template. No amplification products were detected in the negative control groups. Painting probes labelled with Dig-11-dUTP successfully hybridized to mitotic metaphases of *Q. boulengeri* with normal karyotype. We repeated the whole-chromosome painting three times, and the fluorescent signals always appeared on the chromosome 1 pair and not elsewhere in the genome, which demonstrated the accuracy of the mapping results (Fig. 1a). These data indicated that chromosome 1 was sex-associated.

### Painting FISH

Probes of chromosome 1 were hybridized successfully to the metaphase spreads and provided more definitive evidence for reciprocal translocation. The results of FISH showed strong and specific fluorescent signals were present on chromosome 1 and its homologues (Fig. 1). For the normal karyotype (type I), since it contained a pair of homomorphic chromosome 1 and a pair of homomorphic chromosome 6 (MM/mm), the fluorescent signals were detected in the largest pair of homomorphic chromosome 1 (MM); faint signals present on other chromosomes may represent impurities or repetitive sequences incompletely blocked by C_0_t-1 (Fig. 1a). For the translocation heterozygotes (type IV), both pairs of chromosomes 1 and 6 were heteromorphic (MT/mSt), and the fluorescent signals were observed in the largest metacentric chromosome 1 (M), the telocentric chromosome including part of chromosome 1 (T), and a large sub-telocentric chromosome 6 (St, Fig. 1b).

### Sequences analyses of the sex-linked marker B08

Locus B08 contained dinucleotide microsatellite-like repeats, and we found that variation of haplotypes (47 variable sites) existed not only in repeat units (4–6 variable sites) but also in the flanking sequences (41–43 variable sites). In most cases, males presented two haplotypes, which may belong to the X and Y chromosomes; the size difference between these two haplotypes was 27–35 bp. Females showed different situations according to different karyotypes. Females with a normal karyotype had only one haplotype, while females with rearranged karyotypes presented two haplotypes in each individual, with a size difference of 6 bp.

**Fig. 2:**
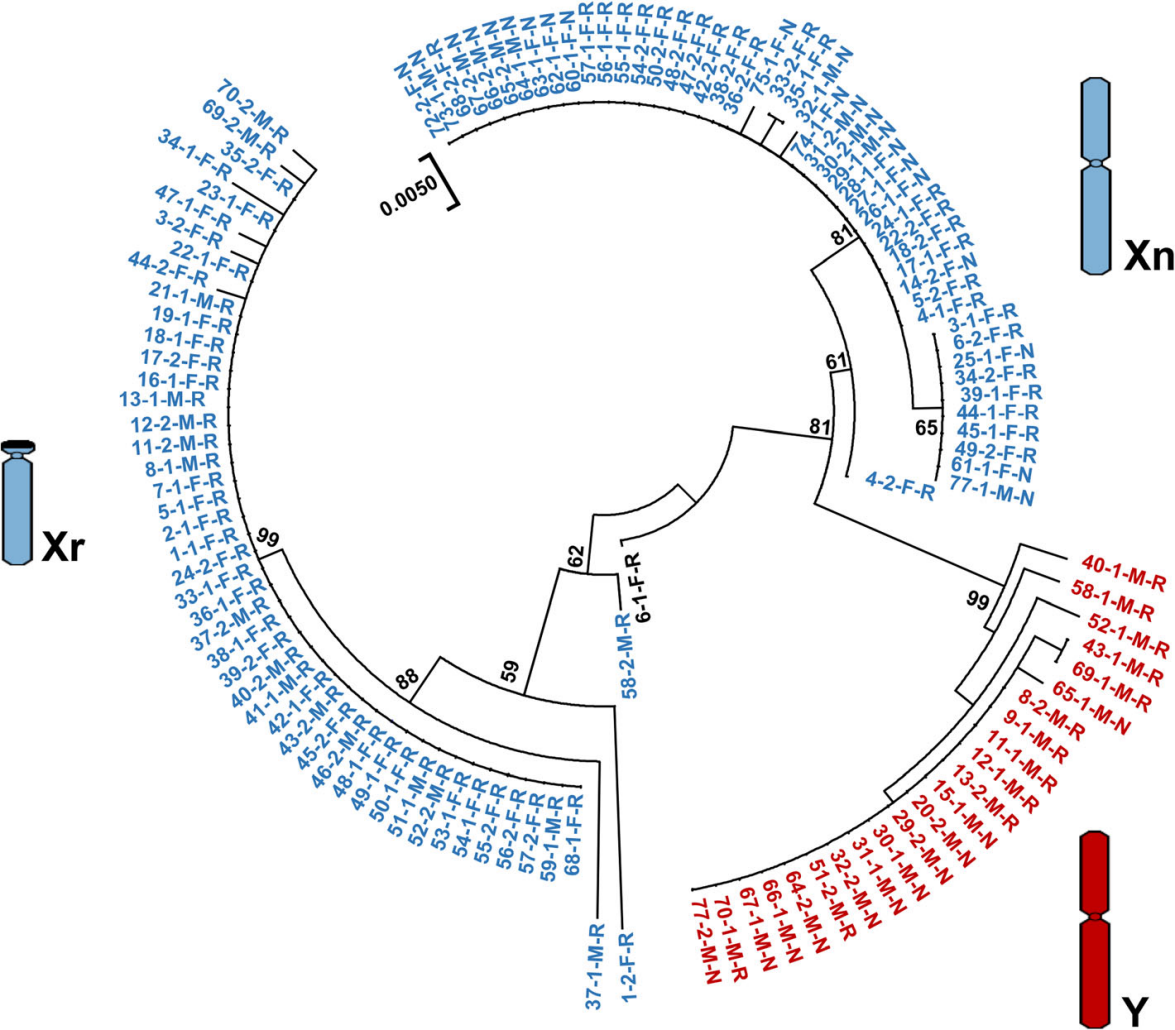
Neighbour-joining tree based on sequences of the sex-linked locus B08. The number at each node is the bootstrap value*.* The element of each sequence name represents the specimen number, the first or second sequence of this specimen, the sex (M for male and F for female) and the karyotype. Note: Xn = normal X chromosome, Xr = rearranged X chromosome

N-J tree demonstrated three distinct clades with strong support nodes (Fig. 2). The sequences in one of the clusters (red) were all from males, suggesting that this cluster corresponded to the male-specific haplotypes and Y chromosome. The other two clusters showed a generally large mix of alleles from males and females, one of which contained sequences of only individuals with rearranged karyotypes, suggesting that it corresponded to rearrangement-specific haplotypes and rearranged chromosomes. The remaining cluster contained sequences of different sexes and different karyotypes (excluding males with rearranged karyotypes), which may represent normal haplotypes in different kinds of individuals. Given that the Y chromosome existed only in males and the X chromosome existed both in males and females, it was possible that reciprocal translocations occurred on the X chromosomes. Three well-supported clades may match up with the Y chromosome, rearranged X chromosome and normal X chromosome (Y, Xr, Xn; Fig. 2).

## Discussion

### Chromosome 1 is the sex chromosome pair

The sex-linked marker was amplified successfully from the DNA of microdissected chromosome 1, indicating a definite assignment to chromosome 1. Specific fluorescent signals of painted FISH were displayed exclusively on chromosome pair 1, which demonstrated the accuracy of the microdissected chromosome 1 and single-cell whole-genome amplification technique. Chromosome 1 was interpreted as the sex chromosome pair in this frog.

Moreover, we found that the sex-linked marker B08 was located on the long arm of chromosome 1. Since the translocation involved the exchange of almost the entire short arm of chromosome 1 [14], all individuals with the rearranged karyotypes would have altered genotypes if the sex-linked marker were located on the short arm. Supposing that the sex-linked marker was on the short arm of chromosome 1, the individuals with type II and V would have three alleles due to having one or two additional segments from chromosome 1, while those with type III would have one allele due to losing a segment of chromosome 1, and those with type IV would still have two alleles due to neither losing nor gaining any segments. However, all individuals with rearranged karyotypes showed two different haplotypes. Therefore, only sex-linked markers located on the long arm of chromosome 1 could explain the pattern of genotypes and sequence haplotypes.

### Translocation occurred on the X chromosome

Why were chromosome heteromorphisms seemingly unrelated to sex at the cytological level? X-chromosome translocation, which caused rearranged heteromorphisms in the natural population, best explained this phenomenon.

Translocations on the sex chromosomes in *Q. boulengeri* were confirmed by whole-chromosome painting. Qing et al. [14] suggested that reciprocal translocation independently evolved once in this species. After the translocation rearrangement randomly occurred in a single individual, the translocation heterozygote (type IV) individual mated with a normal individual (type I) and produced more individuals with rearranged karyotypes. When individuals with rearranged karyotypes mated with each other, those of all five karyotypes could be produced. Our results of whole-chromosome painting verified the single origin of the chromosomal translocation polymorphisms by the evidence of an overall homology in chromosome 1.

**Fig. 3:**
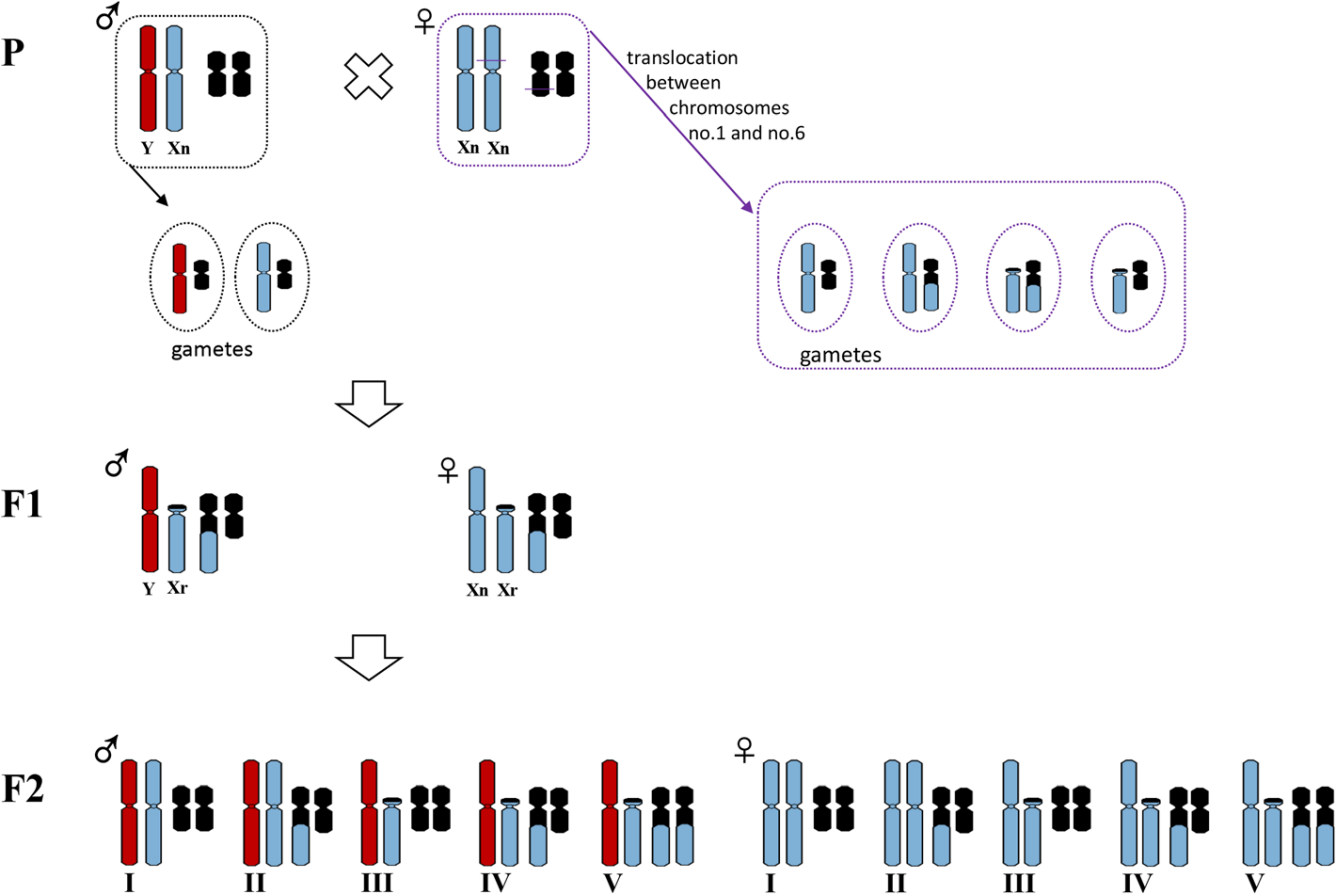
Theoretical karyotypes of progeny after translocations occurred on the X chromosome. The variant heteromorphisms resulted from a reciprocal translocation between chromosomes 1 and 6. Karyomorph type I showed homomorphic chromosomes 1 (M/M) and 6 (m/m); karyomorph type II had homomorphic chromosomes 1 (M/M) and heteromorphic chromosomes 6 (m/St); karyomorph type III was found to possess heteromorphic chromosomes 1 (M/T) and homomorphic chromosomes 6 (m/m); karyomorph type IV had heteromorphic chromosomes 1 (M/T) and 6 (m/St); karyomorph type V was characterized with heteromorphic chromosomes 1 (M/T) and homomorphic chromosomes 6 (St/St)

Supposing a translocation occurred on one of the X chromosomes in females, four different gametes would be produced during meiosis (Fig. 3). As the reciprocal translocation evolved just once, this translocation heterozygote individual (type IV) likely mated with a normal individual (type I) to produce F1 hybrids including both male and female translocation heterozygotes. When these translocation heterozygotes mated with each other, all five karyotypes could be produced in both males and females. In contrast, if a translocation occurred on the Y chromosome, the only male of the translocation heterozygote would have mated with a normal female. In this case, after F1 hybrids hybridized freely or backcrossed, the F2 hybrids would not have displayed all five karyomorphs in both males and females. Theoretically, for females, only two karyomorphs (type I and type II) could be observed if translocations occurred on the Y chromosome, which was not consistent with the five female karyotypes observed in nature.

When the translocation occurred on one of the X chromosomes in females, two kinds of X chromosomes, i.e., normal and translocated X chromosomes, existed in the rearranged populations. Compared with the normal X chromosome (Xn chromosome), the translocated chromosome (Xr chromosome) changed morphologically from metacentric to telocentric, while the Y chromosome remained unchanged. This assumption was supported by phylogenetic inference. Three distinct clades showed a clear pattern of sex chromosome differentiation and represented the unusual sex chromosome system in *Q. boulengeri.* The restriction of genetic recombination could be present between not only the Y and the normal X chromosomes but also the Y and the translocated X chromosomes (Fig. 2). The sex-linked locus, with homologous genes on opposite sex gametologs showing quite different haplotypes, is most likely to be located in the non-recombining region of the sex chromosomes. No female haplotypes were included in the male-specific cluster (Y chromosome), meaning that the sequence divergence and lack of recombination were caused by sex differences. The Y and two types of X chromosomes are differentiated at the sex-linked locus, and there, sex chromosomal dimorphisms emerged in this frog species*.*

### X chromosomes are infrequently derived

In most cases, the Y or W chromosome shows variation in sequence and/or morphology [1–3]. The fragile Y hypothesis predicts that genetic differentiation involving the Y chromosome is more likely to evolve than differentiation involving the X chromosome [23, 24]. For the Y chromosome, the region of suppressed recombination would accumulate sexually antagonistic mutations and lead to genetic differentiation, while the X chromosome recombines normally in females [9]. However, this hypothesis may not apply to all species.

Occasionally, the X chromosome may also have a chance to mutate and present signs of differentiation and/or structural changes. Theoretically, the restriction of genetic recombination is a necessary condition for sex chromosome differentiation [25, 26]. Either X or Y chromosome rearrangement could promote sex chromosome differentiation. In the turtle *Staurotypus salvinii*, the X differs from the Y and from its presumed homologues in closely related species, and this situation was caused by the translocation of a heterochromatic short arm onto the X chromosome [27]. Unlike its closely related species, *Eupsophus roseus*, which presents homomorphic and metacentric sex chromosomes, the sex homologues are heteromorphic in the frog *Eupsophus migueli*; the Y chromosome is metacentric and of the same size as the telocentric X chromosome, and a pericentric inversion may have modified the morphology of the X chromosome [28]*.* In *Q. boulengeri*, our data indicated that the differentiation of two X-chromosome types was caused by a translocation. There were no haplotypes of the normal X chromosome clustered in the X rearrangement-specific clade, pointing to inhibited recombination caused by the translocation rearrangement. Thus, X chromosome rearrangement may also accelerate sex chromosome differentiation. There was no morphological difference between the karyotypes of males and females, suggesting it may represent the early stages of sex chromosome differentiation.

This abnormal X-chromosome translocation may be useful to interpret sex chromosome dynamics. As suppressed recombination of sex chromosomes evolved independently of reciprocal translocation in *Q. boulengeri* [29], the rearrangement shown here should have indirect influence on sex chromosome differentiation. When rearrangements occurred on sex chromosomes, the crossing over between sex chromosomes would be suppressed, making sex chromosomes vulnerable to the operation of forces that lead to a reduction in their effective population size and thus enhancing the power of drift [30, 31]. Without the translocation homozygote, sex chromosomal dimorphisms in *Q. boulengeri* could be in a floating situation, and preserved in small populations through random genetic drift. Expanding the present sex-linked markers (e.g. with RAD-Seq markers) and pedigree analysis would allow better appraisal and quantitative test of the evolutionary forces driving sex chromosome differentiation. As supported by an increasing amount of developmental, demographic and genetic evidence, *Q. boulengeri* may offer an ideal system to investigate the dynamic processes of sex chromosome evolution.

#### Conservation benefits

The species *Q. boulengeri* is a very common frog, widely distributed in low mountainous regions along the edges of Sichuan Basin and nearby areas in central and southern China [32]. With a large body size, this frog has been a commercially valuable species for food consumption for several hundred years. Over-exploitation for human consumption, decreased distribution, habitat destruction and degradation, and water pollution are resulting in the decrease of *Q. boulengeri* [33]. *Q. boulengeri* is listed as “Endangered” species in the 2004 IUCN Red List [34]. Now, it is refreshed as “Vulnerable” species in China Species Red List [35], due to the successful breeding and the effective implementation of government-promoted conservation strategies in recent years.

For effective conservation, we should be aware of the sex-ratios and karyotype-ratios of the individuals selected for breeding. Our research provides useful information on population restoration of *Q. boulengeri*, and the sex-linked marker can be applied to the future breeding program. The sequence variation of the sex-linked marker could be used as a diagnostic tool to identify the heterogametic sex and the rearranged karyotypes in the samples at juvenile stages. Proper analyses of sex-ratios and karyotype-ratios are necessary to the release of captive-bred individuals into the wild, because males and females may contribute unequally to the wild population, as well as chromosome rearrangements may reduce fertility and fitness in this frog [14].

Our results also have important conservation implications for understanding the decline of *Q. boulengeri*, especially in the context of environmental pollutants. Although genetic sex determination (GSD) prevails in amphibians, environmental pollutants would affect its early development and sex determination. Pesticides and anthropogenic endocrine disruptors, for instance, may result in the primary sex ratio biased and sex reversal, which was considered as one of the several potential causes of amphibian decline in recent years [36, 37]. We can identify the heterogametic sex using sex-specific markers to estimate the sex ratio biased and sex reversal, which would be helpful to understand the endocrine-disruptor hypothesis as a likely explanation for the decline of this frog.

## Conclusion

In the present study, we demonstrated that sex chromosomal dimorphisms are caused by X-chromosome translocation, which is rare in nature. Sex chromosomes, including the Y and two types of X chromosomes, are differentiated among haplotypes at a sex-linked locus. The X chromosome occasionally has gone processes of genetic differentiation and/or structure changes, which may promote sex chromosome differentiation. These dimorphic sex chromosomes presenting in both sexes may represent early stages and have the potential to increase our understanding of sex chromosome evolution.

## Additional files

Additional file 1: **Table S1.** Sequence data of the sex-linked marker B08 in *Quasipaa boulengeri*. (XLSX 14.6 kb)
